# Child Maltreatment Is Associated with a Reduction of the Oxytocin Receptor in Peripheral Blood Mononuclear Cells

**DOI:** 10.3389/fpsyg.2018.00173

**Published:** 2018-02-27

**Authors:** Sabrina Krause, Christina Boeck, Anja M. Gumpp, Edit Rottler, Katharina Schury, Alexander Karabatsiakis, Anna Buchheim, Harald Gündel, Iris-Tatjana Kolassa, Christiane Waller

**Affiliations:** ^1^Department of Psychosomatic Medicine and Psychotherapy, Ulm University, Ulm, Germany; ^2^Department of Clinical and Biological Psychology, Ulm University, Germany; ^3^Institute of Psychology, University of Innsbruck, Innsbruck, Austria

**Keywords:** oxytocin, oxytocin receptor, PBMC, child maltreatment, anxiety, attachment

## Abstract

**Background:** Child maltreatment (CM) and attachment experiences are closely linked to alterations in the human oxytocin (OXT) system. However, human data about oxytocin receptor (OXTR) protein levels are lacking. Therefore, we investigated oxytocin receptor (OXTR) protein levels in circulating immune cells and related them to circulating levels of OXT in peripheral blood. We hypothesized reduced OXTR protein levels, associated with both, experiences of CM and an insecure attachment representation.

**Methods:** OXTR protein expressions were analyzed by western blot analyses in peripheral blood mononuclear cells (PBMC) and plasma OXT levels were determined by radioimmunoassay (RIA) in 49 mothers. We used the Childhood Trauma Questionnaire (CTQ) to assess adverse childhood experiences. Attachment representations (secure vs. insecure) were classified using the Adult Attachment Projective Picture System (AAP) and levels of anxiety and depression were assessed with the German version of the Hospital Depression and Anxiety scale (HADS-D).

**Results:** CM-affected women showed significantly lower OXTR protein expression with significantly negative correlations between the OXTR protein expression and the CTQ sum score, whereas plasma OXT levels showed no significant differences in association with CM. Lower OXTR protein expression in PBMC were particularly pronounced in the group of insecurely attached mothers compared to the securely attached group. Anxiety levels were significantly higher in CM-affected women.

**Conclusion:** This study demonstrated a significant association between CM and an alteration of OXTR protein expression in human blood cells as a sign for chronic, long-lasting alterations in this attachment-related neurobiological system.

## Introduction

Stressful life experiences are strongly associated with a higher risk for the development of psychiatric diseases (Agid et al., 2000). An increasing body of literature has specifically focused on the effects of negative early life experiences, termed child maltreatment (CM), on physical (Heim and Nemeroff, 2001) and mental health (Edwards et al., 2003). Experiencing CM has been associated with an increased risk for trauma spectrum disorders such as posttraumatic stress disorder and depression (Felitti Md et al., 1998), while the development of anxiety disorders has been more closely associated with early familial factors like parental loss in childhood (Heim and Nemeroff, 2001; Kendler et al., 2009).

Oxytocin (OXT), a neuropeptide consisting of nine amino acids, is mainly produced in the paraventricular and supraoptic nuclei of the hypothalamus (Insel, 1992). Oxytocinergic neurons project to brain regions involved in social and maternal behavior (Gimpl and Fahrenholz, 2001; Strathearn, 2011). OXT has anxiolytic effects and is related to the reduction of stress behavior (Gimpl and Fahrenholz, 2001; Neumann and Landgraf, 2012). Furthermore, peripheral OXT effects are associated with endocrine and immune functions, like anti-inflammatory effects (Gimpl and Fahrenholz, 2001; Pont et al., 2012). Several animal studies investigated the role of attachment on different central and peripheral peptides. In particular the Brain-Derived Neurotrophic Factor (BDNF) was found as a significant modulator regarding social attachment behavior (Marazitti et al., 2008; Branchi et al., 2013). Over the last 20 years, corresponding investigations in humans on attachment behavior have increased dramatically. Attachment patterns in adults and their infants are strongly associated with emotional regulation (Bowlby, 1969; Ainsworth et al., 1978). To evaluate adult attachment representation mostly self-reports are used (Rochman et al., 2008; Kiss et al., 2011) and only few studies work with narrative interviews to assess attachment, like the Adult Attachment Interview (AAI) or the Adult Attachment Projective Picture System (AAP) (Buchheim et al., 2009). However, several clinical studies confirm the feasibility of the AAP as a stimulus in attachment-related neurobiological science (Buchheim et al., 2006, 2008).

Interpersonal attachment, adverse life experiences and perceived stress were found to be associated with blood levels of OXT (Emeny et al., 2015). OXT plasma levels have been shown to be associated with different attachment representations in humans (Bakermans-Kranenburg and van Ijzendoorn, 2013). A study by Pierrehumbert and Colleagues (2012) investigated plasma OXT before and after a laboratory stress procedure (*Trier Social Stress Test*, TSST) and found higher OXT levels after the TSST stressor only in securely attached individuals (Pierrehumbert et al., 2012). Olff et al. (2013) reported higher plasma OXT levels in adults with CM experiences (Olff et al., 2013). In contrast, a study by Heim and Associates (2009) showed lower levels of OXT in the cerebrospinal fluid of women with a history of CM (Heim et al., 2009). In summary, results on OXT in the central nervous as well as in peripheral organ systems are controversial (Valstad et al., 2016) and, with respect to depression and anxiety, studies about an association between central and peripheral OXT levels yielded inconsistent data (Massey et al., 2016).

The oxytocin receptor (OXTR) is a seven transmembrane domain G-protein, located in the cytoplasmic membrane structure (Gimpl and Fahrenholz, 2001; Zingg and Laporte, 2003), and is expressed in the brain, peripheral tissues as well as in lymphocytes (Gimpl and Fahrenholz, 2001; Yamaguchi et al., 2004). The expression of the *OXTR* gene has already been determined, e.g., in lymphocytes from human peripheral blood and also in macrophages via real-time quantitative PCR (qPCR) (Yamaguchi et al., 2004; Szeto et al., 2008). CM has been shown to be associated with an altered immune function in adulthood (Boeck et al., 2016) and research has provided evidence for an anti-inflammatory role of OXT via binding to its specific receptor (Szeto et al., 2008). Several studies found associations between the OXTR and CM experiences, using methods for the determination of *OXTR* gene methylation which may result in an altered *OXTR* gene expression (Kumsta and Heinrichs, 2013; Smearman et al., 2016). The group of Smearman et al. (2016) reported higher *OXTR* gene methylation associated with CM experiences (Smearman et al., 2016). Additionally, low maternal care was also found to be associated with higher OXTR methylation (Unternaehrer et al., 2012). These findings suggest an important role of the OXTR in understanding the influence of CM on biological processes. However, *OXTR* gene expression or *OXTR* gene methylation study results can be not directly compared with the OXTR protein level on immune cells.

Therefore, we aimed to implement the quantification of OXTR protein expression in human peripheral blood mononuclear cells (PBMC). PBMC fraction consist of different cell types: lymphocytes (T cells, B cells, and NK cells), monocytes, and dendritic cells. In human PBMC, lymphocytes are in the range of 70–90%, monocytes from 10 to 20%, while dendritic cells are rare, with 1–2% (Kleiveland, 2015). In humans, the frequencies of these populations vary across individuals. Recently, OXTR expression via western blotting has been reported for human myometrium during pregnancy (Grotegut et al., 2013) and in bovine lymphocytes (Ndiaye et al., 2008). Applying the same technique, we expected to find a negative association between CM load and OXTR protein expression in PBMC, together with lower OXT levels in peripheral blood plasma. Furthermore, we expected that this negative association would be more pronounced in mothers with CM and insecure attachment representations.

## Materials and methods

### Study design

The study was approved by the Ethics Committee of Ulm University and was conducted in accordance with the Declaration of Helsinki. Written informed consent was obtained from all subjects prior to their participation. Women were recruited at the maternity ward of Ulm University Hospital within 1 week after parturition and were invited to participate at two consecutive time points (t_0_, t_1_). Time point t_0_ was up to 6 days after delivery in the maternity ward of the Ulm University Hospital and consisted of the assessment of basic sociodemographic, medical and childhood-related data (CTQ) (Bader et al., 2009). At t_1_, 3 months postpartum, mothers were invited for a psycho-diagnostic interview at the Clinical & Biological Psychology work group (Ulm University). After a resting phase of ~15–20 min, the attachment representation was assessed with the Adult Attachment Projective Picture System (AAP) (George and West, 2012). Both time points (t_0_ and t_1_) were supervised by trained psychologists. Before the psychological assessment, whole blood samples were collected by venous puncture into EDTA-buffered collection tubes (Sarstedt, Nuermbrecht, Germany) for the Ficoll-based isolation of PBMC. OXT levels were measured in plasma aliquots generated from another EDTA-buffered sample of whole blood immediately before the Adult Attachment Projective Picture System (AAP).

### Participants

In total, 1,460 women were contacted for study participation in the maternity unit of the Ulm University Hospital. Exclusion criteria were age <18 years, insufficient knowledge of the German language, severe complications during parturition or health problems of mother and/or child, premature delivery, current drug consumption, a history of psychotic disorders or current infections. Two-hundred and forty mothers provided written informed consent and completed the screening interview (t_0_). Sixty-seven mothers followed the invitation and participated in the 3-months follow-up interview (t1). Eighteen subjects had to be excluded: five due to insufficient recording quality of the AAP narratives, one because of blood sampling failure, one because of an acute infection, and eleven because of limited availability of biomaterial for western blot analyses. Furthermore, all mothers who breastfed prior to blood sampling (one hour before the AAP starts) were excluded from the biological analyses. One plasma OXT value could not be assessed because of blood sampling failure for one woman of the CM− group. No significant group differences for age and body mass index (BMI) were found. Thus, the final study cohort consisted of *n* = 49 mothers.

### Psychological questionnaires

As mentioned before, the German version of the Childhood Trauma Questionnaire (CTQ; Bader et al., 2009) was used to assess experiences of emotional, physical, or sexual abuse, as well as emotional and physical neglect. The CTQ covers these five subscales with five items each that are rated on a five-point Likert-scale, whereby higher values indicate a higher load of CM experiences. The sum score over all 25 items (ranging from 25 to 125) was calculated as a cumulative measure of maltreatment experiences, i.e., the maltreatment load (Schury and Kolassa, 2012). Cut-off criteria established by Bernstein and Fink (1998) were applied to classify the severity of CM experiences in each of the subscales as “none,” “low,” “moderate,” or “severe” (Bernstein and Fink, 1998). Based on these classifications, the study cohort was split into two groups: those who reported “moderate” or “severe” CM experiences in at least one subscale of the CTQ were categorized as CM+ (*n* = 15), all other subjects were categorized as CM− (*n* = 34). The validated German version of the Hospital Anxiety and Depression Scale (HADS-D) was applied to quantitatively evaluate symptoms of anxiety and depression, which are pooled in two subscales of 7 items rated on a four-point Likert-scale, that can be classified as follows: 0–7 no pathological findings, 8–10 suggestive mood disorder, >11 probable presence of anxiety/depressive disorder (Herrmann-Lingen et al., 1995).

### Attachment measure

The Adult Attachment Projective Picture System (AAP) consists of a set of picture stimuli and is commonly used to assess the attachment representation in adults (George and West, 2012). This picture set includes eight line drawings consisting of one neutral warm-up picture and seven attachment scenes. Attachment scenes show attachment situations, where individuals are alone or in potential attachment dyads. During the AAP, individuals are requested to tell a story related to the situation depicted in each single stimulus. The AAP interview was performed by trained psychologists (trained by coauthor: AB). The reported stories were audio-recorded and recorded verbatim. Further, the stories were documented for the categorization of attachment representations. “Connectedness” and “Agency of Self” are evaluated in the narrative responses to “monadic” pictures, representing a person alone. In contrast, the dyadic picture scenes are judged by their degree of synchrony in the described interactions. A secure attachment classification (F) is coded, when individuals show a high level of connectedness and synchrony. Insecure-dismissing (Ds) or insecure-preoccupied (E) individuals point out an absent or dysfunctional relationship in the AAP evaluation. Finally, individuals with an unresolved trauma (U) are overwhelmed by attachment-related trauma like fear or threat (George and West, 2012). Recent studies revealed inter-rater reliability for the four attachment groups of 90%, κ = 0.84, *p* < 0.001, and for secure and insecure groups even of 97%, κ = 0.89 and a *p* < 0.001 (George and West, 2012).

### Plasma OXT levels

Blood samples were drawn from antecubital veins into 7.5 ml blood monovettes containing EDTA (Sarstedt, Germany). EDTA monovettes and tubes were pre-chilled on ice. Immediately after blood collection, EDTA monovettes were centrifuged at 4°C at 1.300 g for 15 min. A defined volume of 800 μl resulting plasma was aliquoted for the determination of OXT. After finalizing the batch, all plasma samples were shipped on dry ice to the laboratory of R. Landgraf (RIAgnosis, Sinzing). Plasma OXT was measured in extracted plasma samples and determined by a validated radioimmunoassay (RIA) with an assay sensitivity in the 0.1 pg/sample range (Kagerbauer et al., 2013).

### Measurement of OXTR protein expression in human PBMC

A Ficoll-Hypaque gradient centrifugation was performed to isolate PBMC according to the manufacturer's protocol (GE Healthcare, Chalfon St Giles, UK). Immune cells were lysed using standard procedures (Masutomi et al., 2005) and resulting protein containing supernatant was frozen at −80°C and stored for western blotting. Fifteen micrograms of total protein extract was used for further sample preparation. Proteins were separated with SDS-PAGE (sodium dodecyl sulfate polyacrylamide gel electrophoresis), followed by immunoblotting of the separated proteins on a membrane. An internal reference sample was used on each blot, as an internal valid control sample. PVDF-membrane was incubated with primary antibodies [anti-OXTR, 1:1,000, Sigma Aldrich, Germany; anti-Glyceraldehyde 3-phosphate dehydrogenase (GAPDH; loading control antibody), 1:5,000, Thermo Scientific, Germany] overnight at 4°C. Subsequently, blots were incubated with secondary antibodies (anti-rabbit, 1:1,000, Dako, Glostrup, Denmark; anti-mouse, 1:10,000, Invitrogen, Germany) for at least 1 h at room temperature. The molecular weight of OXTR protein is ~43 kDa and the reliable housekeeping protein glyceraldehyde 3-phosphate dehydrogenase (GAPDH) is detected at 36 kDa. Protein bands were analyzed with the Bio-Rad Software Image Lab 5.0 (Bio-Rad Laboratories). OXTR and GAPDH protein expression were represented relatively to the expression level of an internal reference sample for each blot to minimize variations between different blots. OXTR protein expression values were normalized to the loading control GAPDH and represented as % of an internal reference sample run on each blot.

### Statistics

Residuals of data were tested for normal distribution with a Kolmogorov–Smirnov test. Due to a non-parametric data distribution, group comparisons between CM− mothers vs. CM+ mothers, and secure vs. insecure mothers within the whole study cohort were calculated using Mann-Whitney-*U* tests. Furthermore, correlations of plasma OXT and OXTR protein expression with CTQ sum score, anxiety- and depression levels were performed using Spearman-rho correlation analyses. Distribution of CM− vs. CM+ mothers within the CTQ subscales was tested using a Chi-Squared test (Fishers exact test). Statistical tests were performed with α = 0.05. Data analyses were conducted with IBM SPSS Version 23.

## Results

### Descriptive and clinical characteristics

Clinical and psychometric characteristics of the participants are presented in Table 1. Thirty-four mothers were classified as CM−. Fifteen mothers reported a moderate history of CM in at least one subscale (emotional abuse, physical abuse, sexual abuse, emotional neglect and physical neglect) of the CTQ and were therefore classified as CM+.

**Table 1:** Descriptive and clinical characteristics.

	**CM− mothers (*n* = 34)**	**CM+ mothers (*n* = 15)**	***p*-value**
Age (years)	34.06 (±0.86)	31.93 (±1.26)	n.s
BMI (kg/m^2^)	24.88 (±0.92)	23.92 (±0.87)	n.s
HADS anxiety state	4.65 (±0.39)	8.13 (±0.95)	0.002
HADS depression state	2.65 (±0.33)	5.40 (±1.52)	n.s
CTQ sum score	31.85 (±0.86)	56.07 (±4.62)	<0.001
Emotional abuse	0	9	<0.001
Physical abuse	0	5	0.002
Sexual abuse	0	7	<0.001
Emotional neglect	0	10	<0.001
Physical neglect	0	4	0.006
Secure attachment F (n)	9	1	n.s
Insecure attachment Ds, E and U (n)	21	13	<0.001

*Values are presented as mean ± SE and p-values. P-values are calculated with non-parametric Mann-Whitney-U test and Fisher exact test. CM+, women with at least moderate to severe child maltreatment experiences; CM−, women without a history of child maltreatment; CTQ, Childhood Trauma Questionnaire; BMI, body mass index; HADS, Hospital Anxiety and Depression Scale; F, secure; Ds, insecure-dismissing; E, insecure-preoccupied; U, unresolved trauma*.

### OXTR protein expression in human PBMC

**Figure 1:**
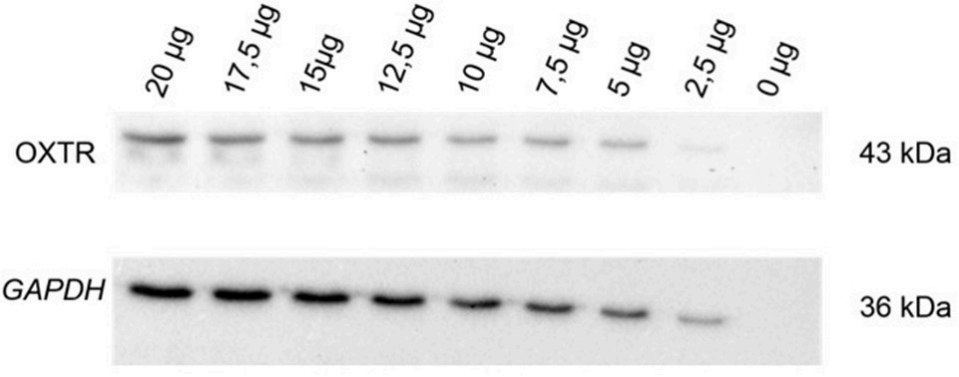
OXTR (43 kDa) expression and corresponding loading control *GAPDH* (36 kDa) were measured to show the linearity in the OXTR antibody detectability in human PBMC. Establishment of the antibody was performed with a protein amount dilution series from 2.5 to 20 μg in 2.5 μg steps within an internal reference control. A protein-free sample (0 μg) was used as negative control; OXTR, oxytocin receptor; *GAPDH, glyceraldehyde 3-phosphate dehydrogenase*; kDa, kilodalton.

In a first step, we used a commercially available antibody (OXTR, Sigma Aldrich, Steinheim, Germany) and tested a series of eight different concentrations of PBMC protein extracts compared to an internal reference control. Protein amount ranged from 2.5 to 20 μg in 2.5 μg steps. A protein free sample was used as a negative control. PageRuler Plus Prestained Protein Ladder (Thermo Scientific, Darmstadt, Germany) was used as a weight reference ladder. Protein series of the OXTR with the corresponding loading control *GAPDH (glyceraldehyde 3-phosphate dehydrogenase)* show a linearity in the antibody detectability shown in Figure 1.

### Plasma OXT levels and OXTR protein expression in relation to a history of CM

**Figure 2:**
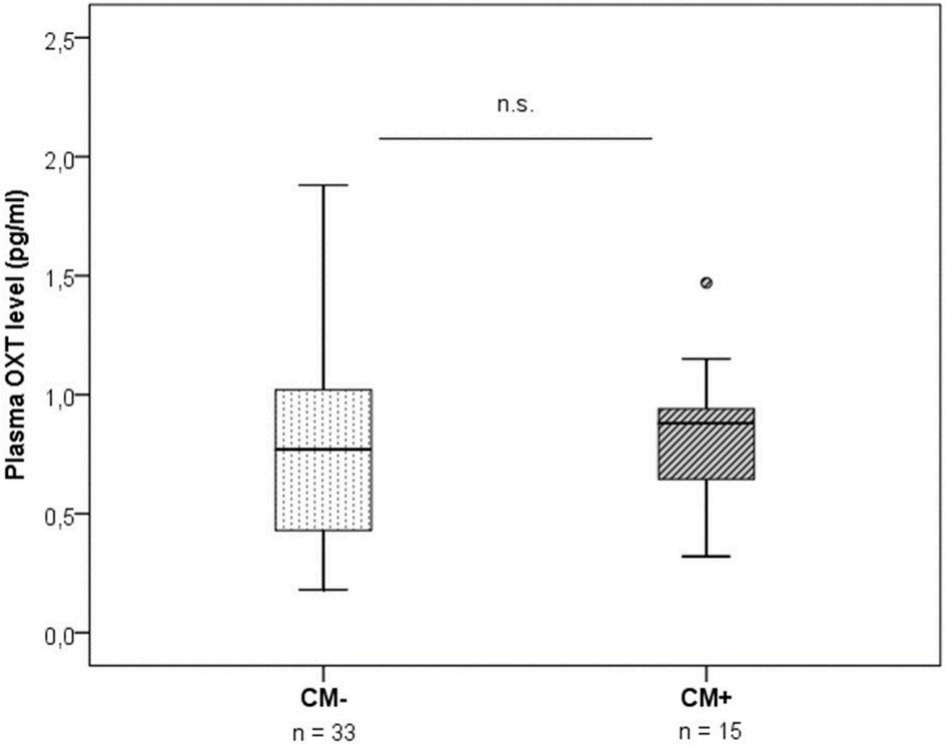
Plasma OXT levels measured by radioimmunoassay in plasma samples of CM− mothers vs. CM+ mothers (*n* = 48). No group differences in OXT were found (Mann-Whitney-*U* test, *z* = −0.49, *p* = 0.625). CM+, women with at least moderate to severe child maltreatment experiences; CM−, women without a history of child maltreatment.

Plasma OXT levels did not differ significantly between the two CM groups (*z* = −0.49, *p* = 0.625) (Figure 2). Furthermore, plasma OXT levels were not significantly correlated with neither the CTQ sum score [*r*_(49)_ = 0.07*, p* = 0.625], nor with the OXTR protein expression [*r*_(49)_ = −0.18, *p* = 0.229].

**Figure 3:**
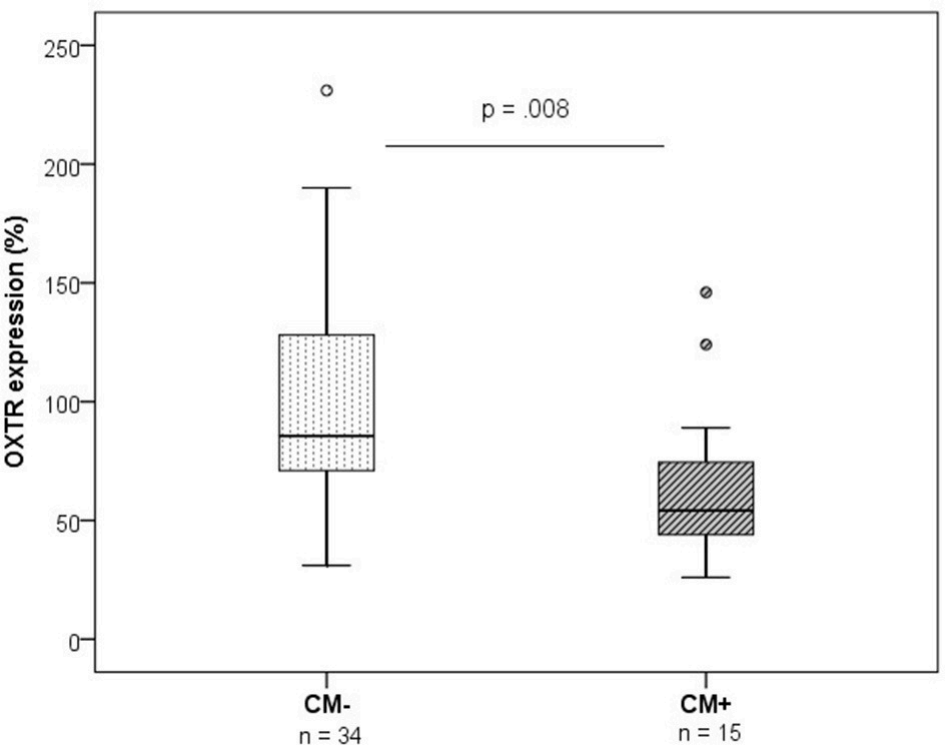
OXTR protein expression levels measured by western blotting (*n* = 49, group means: CM− mothers = 99%, SEM = 8%; CM+ = 66%, SEM = 6%). OXTR protein expression was lower in PBMC of CM+ mothers compared to CM− mothers (*Mann-Whitney-U* test, *z* = −2.66, *p* = 0.008). OXTR protein expression values were normalized to the loading control GAPDH and represented as % of an internal reference sample run on each blot. CM+, women with at least moderate to severe child maltreatment experiences; CM−, women without a history of child maltreatment; SEM, standard error of the mean.

**Figure 4:**
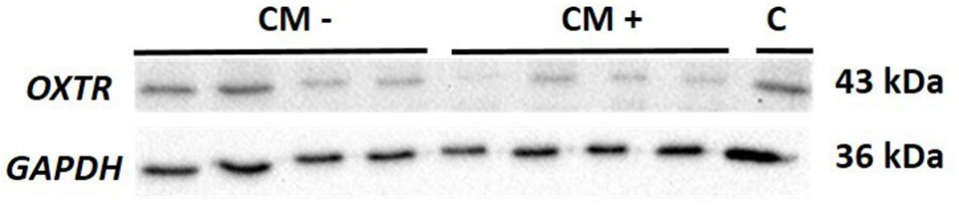
Representative blot of OXTR (43 kDa) protein expression and the corresponding loading control GAPDH (36 kDa) measured in human PBMC. OXTR, oxytocin receptor; GAPDH, glyceraldehyde 3-phosphate dehydrogenase; kDa, kilodalton; CM−, women without a history of child maltreatment; CM+, women with at least moderate to severe child maltreatment experiences; C, internal reference sample running on each blot.

In contrast, OXTR protein expression was significantly downregulated in CM+ compared to CM− women (*z* = −2.66, *p* = 0.008) (Figure 3). A representative blot of *OXTR* protein expression and the corresponding loading control *GAPDH* measured in human PBMC is shown in Figure 4.

**Figure 5:**
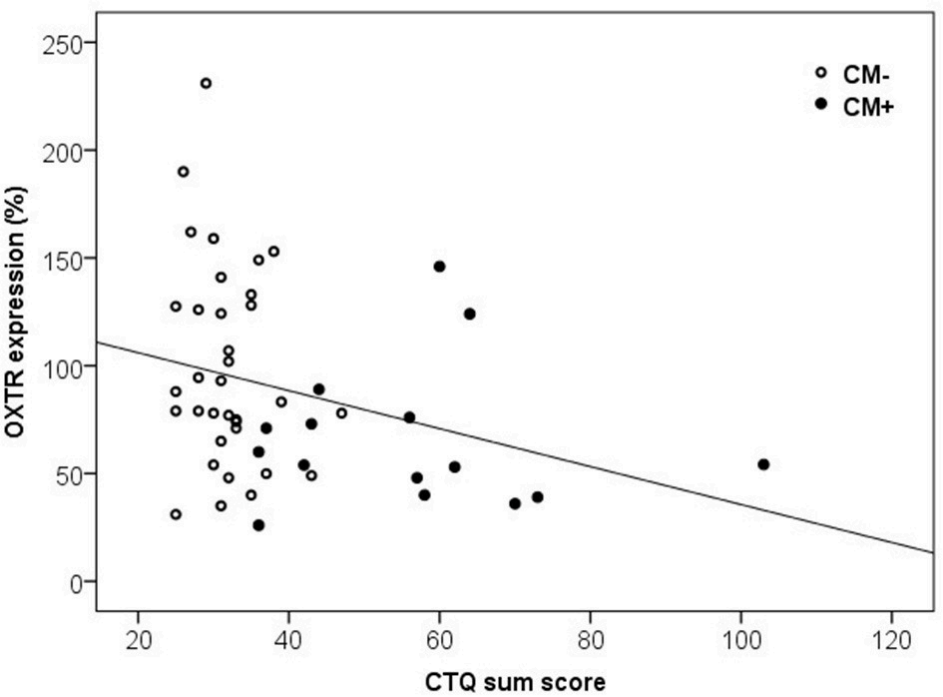
Correlation of CTQ sum scores and OXTR protein expression in human PBMC. Mothers with a higher CTQ sum score presented a lower level of OXTR protein expression in PBMC [Spearman-Rho Correlation, *r*_(49)_ = −0.36, *p* = 0.010]. OXTR, oxytocin receptor; CTQ, Childhood Trauma Questionnaire.

Furthermore, the maltreatment load and OXTR protein expression in PBMC were significantly correlated. We found a negative association between the CTQ sum score and the levels of OXTR protein expression [Spearman-rho Correlation, *r*_(49)_ = −0.36, *p* = 0.010, Figure 5].

### Plasma OXT and OXTR protein expression in relation to symptoms of depression and anxiety

Individuals with CM showed significantly higher anxiety levels compared to CM− mothers (*z* = −3.10, *p* = 0.002). Furthermore, reported anxiety levels were significantly higher with increasing CTQ sum scores [*r*_(49)_ = 0.33, *p* = 0.019]. Severity of depressive symptoms, however, did not differ significantly between the CM+ and the CM− group (*z* = −1.07, *p* = 0.287), nor did the CTQ sum score correlate with the HADS depression sum score [*r*_(49)_ = 0.14, *p* = 0.338]. Similarly, no significant correlations between OXT levels and HADS depression sum score [*r*_(48)_ = −0.028, *p* = 0.849] or HADS anxiety sum score [*r*_(48)_ = 0.03, *p* = 0.829] were found. Finally, OXTR expression in PBMC did neither correlate with the mother's anxiety levels [*r*_(49)_ = −0.07, *p* = 0.641] nor with depression levels [*r*_(49)_ = −0.12, *p* = 0.415].

### Plasma OXT and OXTR protein expression and attachment representations

**Figure 6:**
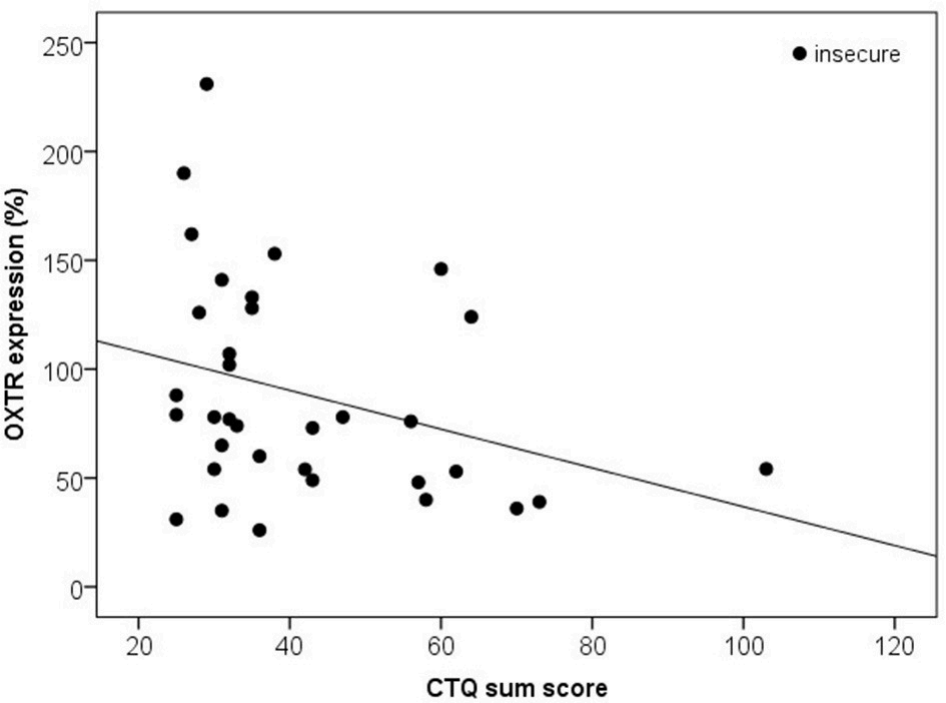
Correlation of the CTQ sum score with the OXTR protein expression in human PBMC of insecure mothers (Ds, E, and U). Insecure mothers revealed lower OXTR protein expression associated with higher CTQ sum scores [Spearman-Rho Correlation, *r*_(34)_ = −0.35, *p* = 0.040]. OXTR, oxytocin receptor; CTQ, Childhood Trauma Questionnaire.

Based on the AAP, 10 mothers were classified as secure (F) and 34 women as (Ds, E and U). Secure compared to insecure mothers did not differ in the HADS anxiety sum score (*z* = 0.00, *p* = 1.000), HADS depression sum score (*z* = −0.03, *p* = 0.977), or in the CTQ sum score (*z* = −1.15, *p* = 0.250). Furthermore, plasma OXT levels did not differ significantly between the secure and the insecure mothers (*z* = −1.55, *p* = 0.121) and there were no significant differences between both attachment groups with respect to OXTR protein expression (*z* = −1.38, *p* = 0.166). Interestingly, the AAP attachment classifications showed, however, significant correlations with the OXTR protein expression in human PBMC depending on the CTQ sum scores: In mothers with insecure attachment representation, lower OXTR protein expression were associated with higher CTQ sum scores [*r*_(34)_ = −0.35, *p* = 0.040], whereas in mothers with secure attachment representation, no association between OXTR protein expression and the CTQ sum scores was found [*r*_(10)_ = −0.48, *p* = 0.160; Figure 6].

## Discussion

OXTR protein expression were significantly lower in CM+ compared to CM− mothers and maltreatment load correlated negatively with OXTR protein expression in PBMC. Additionally, a lower OXTR protein expression was particularly pronounced in women with insecure attachment representations. Furthermore, no associations between OXT levels, CM experiences, depression scores or attachment representation were found, suggesting alterations on the OXTR level but not the peripheral OXT level as a long-term consequence of CM exposure. In accordance with previous findings, this study found a positive correlation between anxiety scores and CM load (Simon et al., 2009), but no association of plasma OXT or OXTR protein expression and anxiety- or depression scores, respectively.

### Peripheral OXT levels in relation to a history of CM

This study found no significant alteration of peripheral OXT levels depending on maltreatment load which is consistent with a study of Chatzittofis et al. (2014). In contrast, several previous studies reported lower or higher levels of peripheral OXT in association with CM experiences (Opacka-Juffry and Mohiyeddini, 2012; Olff et al., 2013; Seltzer et al., 2014). Reasons for the inconsistency in study findings might be 1) the heterogeneity of the methodological spectrum of OXT determination, and 2) the short half-life of the peripheral peptide (1–2 min) (Gimpl and Fahrenholz, 2001; Massey et al., 2016). Our measured plasma OXT levels spread in a physiological range arguing for a reliable RIA assay procedure and were comparable with peripherally measured plasma OXT values in the literature (Marazziti et al., 2006). The used RIA assay determined OXT levels in extracted plasma samples, reflecting a highly validated measuring method (Szeto et al., 2011; McCullough et al., 2013). In this study, plasma OXT levels were assessed at baseline without a specific stimulus that may activate the OXT system. Previous studies observed that maltreated individuals showed a higher OXT release in response to a psychological stressor (Seltzer et al., 2014). In contrast, our own studies also showed no CM-affected plasma OXT alterations, but a significant increase in peripheral OXT levels in response to an attachment stressor (AAP) (Krause et al., 2016). Therefore, the OXT plasma level of maltreated participants might overreact in response to an acute stressor, while it is unchanged with respect to OXT levels in a resting condition.

### OXTR protein downregulation in relation to a history of CM

To the best of our knowledge, this study is the first to determine the OXTR protein expression in human PBMC using western blot analysis. Detection of *OXTR* gene expression using qPCR has already been performed in human blood lymphocytes (Yamaguchi et al., 2004). The regulation of OXTR protein expression is complex (Gimpl and Fahrenholz, 2001) and, until now, a direct link between *OXTR* gene expression levels and OXTR protein could not be found. Group comparisons in our study revealed a lower OXTR protein expression in women with CM experiences. Furthermore, we found a negative association between OXTR protein expression and maltreatment load. These results argue for the sensitivity and variability of the OXTR system in immune cells and suggest long-term alterations especially affecting the OXTR in immune cells. These alterations may occur in a dose-dependent and chronic manner since they can be detected several years up to decades after the exposure to CM. Animal studies have shown an age-related association of early life stress and OXTR binding in the brain of male adult rats. OXTR binding was significantly lower in the lateral septum and the caudate putamen after maternal separation during childhood (Lukas et al., 2010). Supporting our results, Smearman et al. (2016) found a higher methylation of the *OXTR* gene in leukocytes of abused children (Smearman et al., 2016). These epigenetic modifications, i.e., increased DNA methylation, may translate into the downregulation of the OXTR protein expression depending on the maltreatment load. In sum, early life adversity might thereby imprint important long-term effects on signaling cascades of the OXT/OXTR system. Especially, the OXTR downregulation may result in a higher susceptibility for e.g., inflammatory processes or an exacerbated vulnerability to diseases in CM-affected mothers.

### Immunological role of an OXTR downregulation

CM experiences are linked to an altered immune function in adulthood (Boeck et al., 2016). Considering the cell subpopulations of PBMC and their stress-related changes, studies provide evidence for an adaptive alteration in the composition of immune cell subsets following traumatic stress exposure (Sommershof et al., 2009; Morath et al., 2014). Using fluorescence activated cell sorting (FACS), Boeck et al. (2017) found no significant differences between CM+ and CM− mothers regarding the percentages of selected leukocyte subsets CD3+ T cells, CD3+ CD8+ cytotoxic T cells, CD14+ monocytes, CD3− CD14− B and NK cells). These findings strengthen the perspective that our findings of alterations in the OXTR expression are not related to compositional changes in PBMC (Boeck et al., 2017). Individual functions of the OXTR on different sub cells of PBMC are still unknown. Future research should consider the adaptive nature of PBMC composition in the context of CM and the consequences on OXT signaling to further understand the precise nature of our findings. Recently, evidence has accumulated that individuals with CM show an increased inflammatory signaling as reflected by increased levels of pro-inflammatory cytokines such as interleukin 6 (IL-6) and tumor necrosis factor (TNF) (Carpenter et al., 2010; Lopes et al., 2012). Research has provided evidence for an anti-inflammatory role of OXT, as it mediates anti-inflammatory actions via binding to its specific receptor, thereby attenuating the release of pro-inflammatory cytokines (Szeto et al., 2008). Accordingly, OXTR activation stimulates e.g., the expression of several cytokines (IL-2 and IL-4 genes) involved in anti-inflammatory immune responses and attenuates the secretion of pro-inflammatory cytokines (Pont et al., 2012; Oliveira-Pelegrin et al., 2013; Wang et al., 2015). On this account, physiological immune processes like the release of anti-inflammatory cytokines may be impaired after CM exposure, caused by the lower OXTR expression found in individuals with CM experiences. This fact may account for a pro-inflammatory phenotype in adulthood associated with CM experiences. Finally, this study highlight the important issue of further studies, regarding the adaptive immunomodulation and the OXTR system coupled with experiences of early adversity. One possible step could be the investigation of OXTR protein expression in different subsets of PBMC.

### Plasma OXT and OXTR protein expression levels and attachment representation

In literature, the *OXTR* gene was observed as one possible source of variations in infant attachment representation revealing an association of OXTR rs2254298 with infant attachment security (Chen et al., 2011). In our study, the OXTR protein expression was negatively related to maltreatment load especially in mothers with insecure attachment representations. However, we found no association between attachment representation and peripheral plasma OXT levels. Our study cohort comprised 10 secure vs. 34 insecure mothers and therefore we had an unbalanced group distribution. Individuals with a secure attachment representation revealed a higher level of agency (like internalized secure base, mentalizing capacities) and connectedness, whereas insecurely classified individuals showed these capacities to a lower extend (George and West, 2012). Therefore, mothers with an insecure attachment representation may not be able to reflect on attachment-related experiences in a constructive way and to rely on internal resources on a representational level. This could explain why these mothers might be more vulnerable with respect to CM, which may result in lower OXTR protein expression. However, this association should be interpreted with caution due to the relatively small sample size. In contrast to studies showing an effect of the attachment representation on the OXT/ OXTR system, previous studies from our group revealed opposite results with no attachment-depending effects of baseline or plasma OXT levels in mothers measured immediately after the AAP (Krause et al., 2016). Therefore, studies investigating the OXT/ OXTR system in securely and insecurely attached individuals found inconsistent results, especially if peripheral plasma OXT levels were determined. According to our results, the OXTR protein expression may serve as a long-term attachment-related marker.

In conclusion, we provide first results on the measurement of OXTR protein expression in human PBMC. OXTR protein quantification in PBMC may be a promising tool to investigate and to extend our understanding of the role of the OXT/OXTR system on the regulation of immune cell functioning, inflammation and stress reactivity in the context of CM. Following maltreatment experiences during childhood, OXTR protein expression seems to be downregulated in the sense of a “maltreatment scar” that is observable even decades after CM exposure. Therefore, further studies considering the OXTR protein expression in cell subpopulations of immune cells may clarify physiological immune processes like the release of anti-inflammatory cytokines in a more detailed way. The assessment of OXTR in PBMC might be a new biomarker target in attachment and trauma-related research.

## Author contributions

I-TK, HG, AB, CW, and AK conceptualized the study design. Western blot analyses were designed by SK, CB, and AG together with AK. AG performed the protein expression analyses and raw data acquisition. AAP interviews were performed by KS and were classified by AB. Biological data collection and interpretation of the results was performed by SK, CB, and AG with essential support of CW, I-TK, and AK. Statistical data analyses were performed by ER and SK. SK wrote the first draft of the manuscript and edited its final version together with CW. All authors critically revised the manuscript for important intellectual content.

### Conflict of interest statement

The authors declare that the research was conducted in the absence of any commercial or financial relationships that could be construed as a potential conflict of interest.
